# A Fabricated Force Glove That Measures Hand Forces during Activities of Daily Living

**DOI:** 10.3390/s22041330

**Published:** 2022-02-09

**Authors:** Edward F. Austin, Charlotte P. Kearney, Pedro J. Chacon, Sara A. Winges, Prasanna Acharya, Jin-Woo Choi

**Affiliations:** 1 School of Electrical Engineering and Computer Science, Louisiana State University, Baton Rouge, LA 70803, USA; eausti6@lsu.edu (E.F.A.J.); ckearn5@lsu.edu (C.P.K.); pchaco2@lsu.edu (P.J.C.); 2 School of Kinesiology, Louisiana State University, Baton Rouge, LA 70803, USA; sara.winges@unco.edu (S.A.W.); prasanna.acharya@ic.edu (P.A.); 3 School of Sport and Exercise Science, University of Northern Colorado, Greeley, CO 80639, USA; 4 Department of Biology, Illinois College, Jacksonville, IL 62650, USA; 5 Center for Advanced Microstructures and Devices, Louisiana State University, Baton Rouge, LA 70803, USA

**Keywords:** force glove, activities of daily living, hand force, FlexiForce sensor, flexible sensors

## Abstract

Understanding hand and wrist forces during activities of daily living (ADLs) are pertinent when modeling prosthetics/orthotics, preventing workplace-related injuries, and understanding movement patterns that make athletes, dancers, and musicians elite. The small size of the wrist, fingers, and numerous joints creates obstacles in accurately measuring these forces. In this study, 14 FlexiForce sensors were sewn into a glove in an attempt to capture forces applied by the fingers. Participants in this study wore the glove and performed grasp and key turn activities. The maximal forces produced in the study were 9 N at the distal middle finger phalanx and 24 N at the distal thumb phalanx, respectively, for the grasp and key turn activities. Results from this study will help in determining the minimal forces of the hand during ADLs so that appropriate actuators may be placed at the appropriate joints in exoskeletons, orthotics, and prosthetics.

## 1. Introduction

The movie scene in which Luke Skywalker tests out his prosthetic limb is captivating. The device allows him to manipulate objects, but also appears to provide sensory feedback regarding the movements. As sensor technology becomes smaller and more advanced, it allows for a greater understanding of wrist and hand kinematics. Force and motion capture data knowledge of the wrist and hand during ADLs are imperative for building algorithmic biomimetics for human artificial intelligence. These algorithms can be utilized to model prosthetics/orthotics, prevent workplace-related injuries, and understand movement patterns that make athletes, painters, typists, dancers, and musicians unique and elite. Manual muscle testing is the typical universal method used clinically to test force produced by the wrist [1,2,3]. However, force measurement has always been difficult to accomplish in the hand due to the numerous joints of the hand and wrist, small anatomical size, and large degree of movement [4]. Therefore, a strain gauge, referred to as a dynamometer, was introduced in the 1950s [5,6,7]. Multiple studies have been completed to build a library of normative grip strength measurements [8]. This device also has its limitations as it is only able to measure the gross grasp of the hand as a whole. In order to better understand the hand, it is imperative to record individual phalangeal forces and capture motion data during activities of daily living (ADLs).

With the advent of virtual reality and ongoing technology breakthroughs in sensors, there is a focus on creating and utilizing smaller sensors that provide force and motion capture data related to the hand. Asakawa et al. utilized a finger pressure on a touchscreen device to capture maximal forces as high as 6.7 N when performing a stretching function on the touchscreen. However, this activity required multiple fingers, and the resultant actual force per finger (not phalanx) was only 1.02 N [9]. Fabrication of sensor gloves [10,11], electronic skins [12,13], and optical fingernail plethysmography color pressure sensors [14] have been undertaken with the proposed idea of capturing finger biometrics such as force and temperature. However, most of these devices have only been calibrated and never actually utilized during ADLs. A novel study by Kargov et al. utilized 20 force sensing resistors (FSR) dispersed across each finger phalanx and the palm of the hand [15]. This study required individuals with intact and amputated upper extremities to grasp a spherical object. The maximum individual sensor force was 3.8 N for the intact hands compared to 24 N for the prosthetic hand.

A more robust attempt of capturing hand biometrics was termed the scalable tactile glove (STAG). This attempt utilized a dense array of small force-sensing resistive sensors (548) in the palm and fingers of the hand to create a library of various postures of the hand [16]. The working range of the sensors ranged between 30 mN and 0.5 N. Due to the small working ranges, this device would be limited in recording larger forces focused on small areas such as picking up a small pin or grasping heavily weighted objects at a workplace or in a gym. The authors also chronicled the difficulty of variability with forces depending on skin stiffness, changes in skin stiffness with changes in finger posture, and changes in the position of the sensors with changes in finger posture. Some research has focused on motion capture of the fingers in an interactive virtual reality environment to provide feedback about the success of various grasps. The GESTO (glove for enhanced sensing and touching) is one such device that utilizes eleven inertial motion sensors/magnetometers distributed over the dorsum of the fingers and the metacarpals [17]. While the user manipulates virtual objects, the device captures hand motion data. This system is a closed-loop system and provides sensory feedback about the grasp of virtual objects through a vibrotactile motor placed at the fingertips. While this device is a good concept to capture movement and provide feedback in the virtual world, it does not capture forces applied by the hand on real-world objects. For a more in-depth understanding of the current sensor gloves on the market or under research, refer to the literature review performed by Caeiro-Rodrguez et al. [18].

The goal of this experiment was to capture forces on each phalanx during ADLs with minimal set-up after the initial calibration of the device. Questions were raised regarding why not simply apply sensors to the devices to be grasped. First of all, this would require a large assortment of sensors if each tested object was to be covered with sensors. That would be costly and require a significant amount of time to calibrate each of the sensors to each of the objects prior to testing. The concept of using this glove is the ability to switch between objects (and users) without having to calibrate the sensors, as well as the ability to capture the individual forces provided by each of the phalanges. Larger diameter sensors would not give information specific to the individual phalanges of the hand. Instead, there would be more of a gross grasp recording similar to a handheld dynamometer as previously mentioned. Smaller sensors distributed along an object would be more difficult to fit on an object secondary to the tails of the sensors, impeding the ability to place more sensors in closer proximity. If sensors were placed on the objects, the participants would have to be “coached” on how to grab the object in order to place the hands on the sensors to get the best reading. The concept of this experiment was to allow the participants to grasp the device freely and without instruction. An understanding of common hand grasps and forces amongst the participants could assist in developing algorithms that might be used in developing biomimetic algorithms for exoskeletons, prosthetics, and orthotics. It may also provide insight into commonalities amongst elite athletes, musicians, craftsmen, etc. In order to accomplish this goal, this work uses FlexiForce sensors (Tekscan, Inc., South Boston, MA, USA) placed at each of the 14 phalanges to capture forces produced by the hand while performing ADLs.

## 2. Methods and Materials

**Figure 1:**
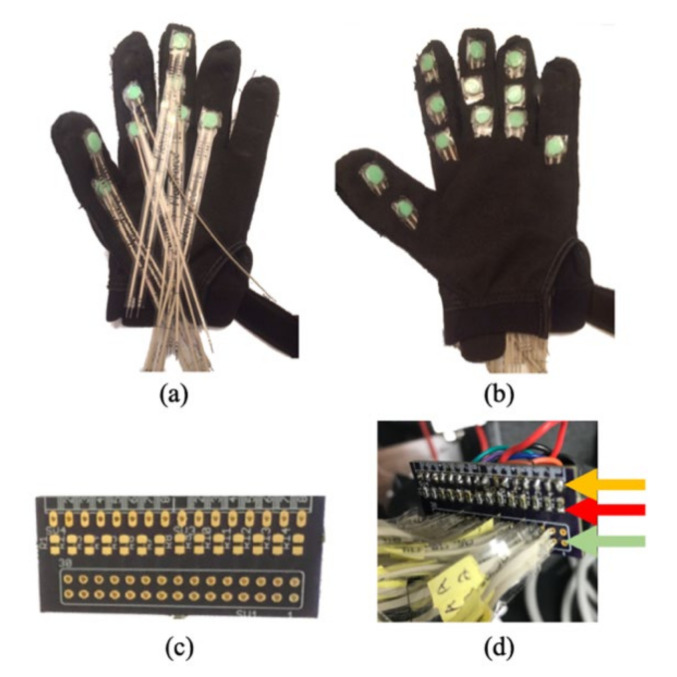
Fabricated force measurement glove: (**a**) glove with FlexiForce sensors; (**b**) glove with FlexiForce sensors and membranes viewable; (**c**) printed circuit board (PCB) to interface the sensors; (**d**) PCB with attachments of FlexiForce sensors (green arrow), 1 MΩ resistor (red arrow), and output wire (orange arrow).

**Figure 2:**
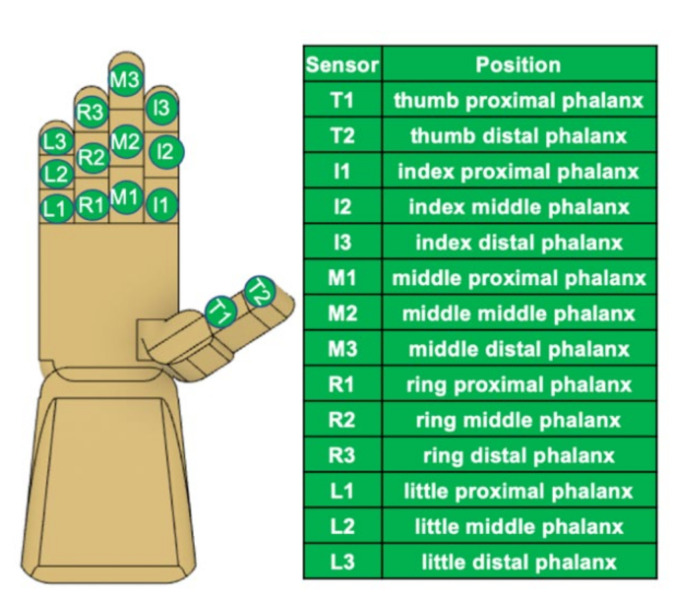
Force sensor positions. A total of 14 FlexiForce sensors were used on each digit and accordingly labeled.

Fourteen FlexiForce A201 standard sensors were used to capture the individual forces for each of the phalanges. These are piezoresistive sensors that demonstrate decreased resistance with increased pressure. FlexiForce A201 has a circular sensing area of 0.375-inch in diameter. Due to the large size of the sensors, each of the sensors were sewn into a standard fabric glove on the palmar side of the hand. A small round thermoplastic membrane was adhered, palmar side down, onto the FlexiForce sensors in an effort to center the forces onto the sensors and prevent the sensors from sinking into the skin. In order not to limit movement, thermoplastic membranes did not cross the joints. The membrane was cut to 0.375-inch in diameter and 0.0625-inch in thickness so that the full diameter of the FlexiForce sensor was utilized. Each of the sensors’ distal pins was soldered parallel to a printable circuit board (PCB) with a 1 MΩ resistor. Output wires were soldered to the PCB and attached on the opposite end to a channel connector provided by Motion Labs Systems (Motion Lab Systems, Inc, Baton Rouge, LA, USA). The channel connectors consisted of eight ports that were attached to the Codamotion (Codamotion, Rothley, United Kingdom) hard drive. A 5 V supply voltage, *V*_0_, was provided by an Arduino microcontroller board parallel to all force sensors. Figure 1 shows the fabricated force measurement glove, and Figure 2 illustrates the defined positions of the force sensor.

**Figure 3:**
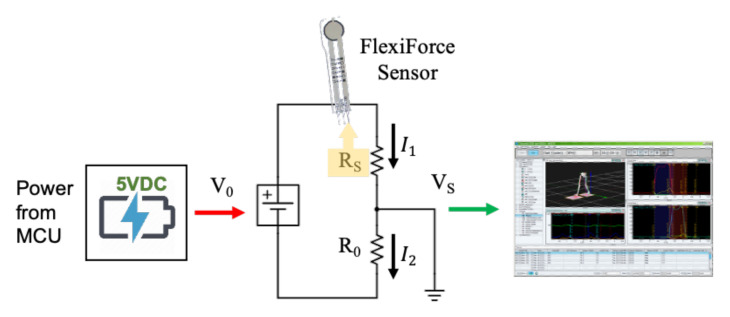
FlexiForce sensor interface with the Codamotion system to transfer all 14 sensor readings.

A voltage divider circuit was created with a reference resistor, *R*_0_, having a value of 1 MΩ in series with a FlexiForce sensor, *R*_S_, as depicted in Figure 3. The sensor voltage, *V*_S_, then will be *V*_S_ = [*R*_0_/(*R*_0_ + *R*_S_)]·*V*_0_. The FlexiForce sensor (*R*_S_) has a resistance greater than 10 MΩ when there is no pressure applied to the sensor. Therefore, we can infer that when there is no pressure applied to the sensor, the denominator approaches infinity (∞). From this, we can deduce that *V*_S_ ≈ 0 V. When the maximum pressure is applied to the sensor, the resistance, *R*_S_, approaches 0, and the subsequent voltage across the sensor becomes *V*_S_ ≈ *V*_0_ = 5 V. Since the force glove was connected to the Codamotion system, there was an internal analog-to-digital conversion. Instead of voltage or resistive measurements, an arbitrary set of numbers were associated with changes in resistance. This necessitated the calibration of those Codamotion numbers with a standardized set of forces applied to the FlexiForce sensors.

## 3. Experimental Results

### 3.1. Force Glove Calibration with Codamotion System

**Figure 4:**
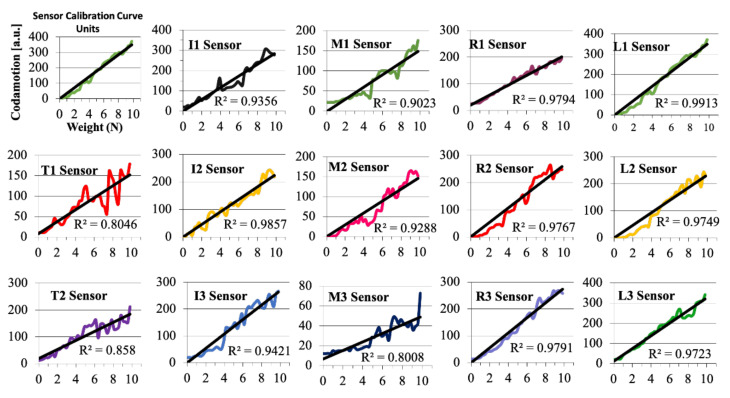
Calibration curve of each sensor embedded in the force glove.

The force data collected from the Codamotion system was in a digital format with an arbitrary zero point. Therefore, a standardized calibration of the sensors was performed prior to any application of the glove to the hand. The intention of the calibration was to correlate a specific force applied to the sensors with the appropriate Codamotion numeric value. In order to accomplish this, each individual sensor was tested after it had been sewn into the force glove. During calibration, the sensor was attached to a three-dimensional (3-D) printed pedestal, while a Winware calibration weight ranging from 0 to 1000 g was applied to each sensor. Datasheets provided by Tekscan revealed that the FlexiForce sensors provide linear conductance [19]. While applying the weights to the sensors, the Codamotion system was utilized to capture each of the forces. The method consisted of applying a 25-g weight, capturing a measurement over a 3–5 s period, and repeating the procedure in increments of 25 g until 1 kg of force was reached. The data were collected at a sample rate of 200 Hz per recording. These files were saved into a.c3d format and then initially analyzed in MATLAB. The recordings occasionally consisted of short regions where many or even all of the sensors gave a zero reading (10 row increments or less). Therefore, those zero points were isolated, and a moving average was utilized to fill in those voids so that the overall average was not skewed. After this averaging, each weight recording was averaged for each of the sensors and the values of Codamotion readings (y-axis) were correlated to the weight applied to the sensor (*x*-axis) in Figure 4 as a calibration curve. Calibration curves reveal that the Codamotion signal per force for each sensor was relatively linear. *R*^2^ values of sensors T1, T2, and M3 were slightly deficient compared to the other eleven sensors. However, those *R*^2^ values were still relatively acceptable at 0.8 or greater, which allowed us to use the glove throughout the experiments. Those sensors remained in the same places (the middle phalanx sensor remained on the middle phalanx) throughout all participants and trials.

Variability between FlexiForce sensors was also evident from the analysis. As can be seen in the graphs of Figure 4, Codamotion maximum values ranged between 400 units and 100 units when nearly 9.8 N of force was applied to the various sensors. These sensors have been known to demonstrate some variability based on temperature fluctuations. However, this experiment was performed in the same laboratory in nearly the same spot. There was an active effort to maintain room temperature during the experiment so as to limit variability. The y-intercept values in the calibration would later be used to correlate force measurements in the can grasp and key turn tasks from Codamotion units.

### 3.2. Force Glove Used during Activities of Daily Living (ADL)

**Figure 5:**
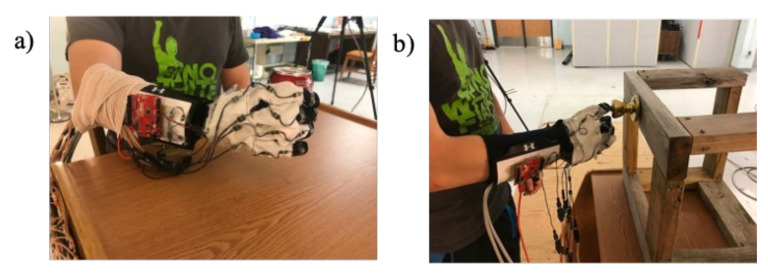
Force glove test setups for (**a**) can grasp and (**b**) key turn activities.

Since the focus was to measure hand force during ADLs, this experiment focused on two ADLs: can grasp and key turning. In these two motions, it was hypothesized that the participants would utilize a spherical gross grasp and lateral pinch grasp, respectively, for each of the activities. Before beginning this experiment, Institutional Review Board (IRB) approval was obtained through Louisiana State University. Three participants voluntarily chose to participate in the experiment and completed a consent form affirming that choice. The participants completed a small questionnaire that obtained information regarding age, race, sex, and prevalence of previous hand injury. Participants ranged between the ages of 21 and 40, were all male, right-handed, and experienced no significant orthopedic injuries, neuromuscular conditions, or medical problems that affected hand motion. The participant then donned the standard size force glove for adults and checked the fit. Prior to beginning the experiment, an experimenter applied manual force to each force sensor while another experimenter monitored the Codamotion output values. This procedure was performed prior to testing with each participant to confirm that each of the sensors was connected to the Codamotion system. Once this preliminary testing was completed, the focus was then turned toward the can grasp and key turn tasks, as shown in Figure 5.

In the can grasp task, the participant was asked to grab a full 12-ounce soda can, lift the can toward his or her mouth (but not touch the mouth), and return the can to the table. This activity was performed three times while being recorded on the Codamotion system. Next, the participant took part in a key turn activity. Here, a door handle with a key lock was secured to a wooden frame so that this activity could be performed in front of infrared/near-infrared cameras without obstruction. In this activity, the participant was instructed to grab a key that was inserted into the lock, turn the key as far as the lock allowed, and then return to neutral. As with the can grasp activity, the participant was asked to repeat this activity three times while being recorded on the Codamotion System.

**Figure 6:**
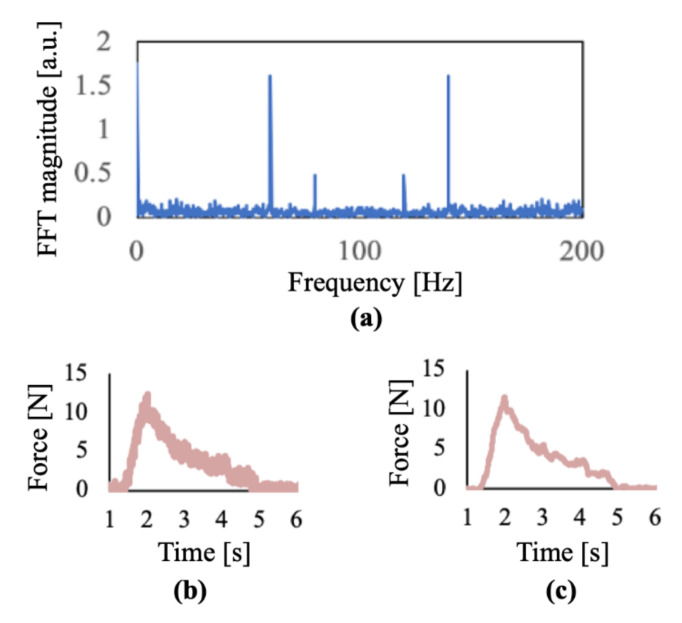
Calibration of can grasp and key turn activities: (**a**) fast Fourier transform of force glove sensor revealing signal noise at 60 Hz and 80 Hz; (**b**) unfiltered sensor data from M3 (middle distal phalanx); (**c**) signals after applying low pass filter (10 Hz). The curve starts inclining when the activity occurs in the graphs.

Upon analysis of the data, it became evident that a different form of calibration was required to truly capture the dynamic motion of the hands. As can be seen in Figure 6, from the M3 sensor, there was still a significant amount of noise in the signal that appeared unrelated to human movement (50–60 Hz AC signals, systolic and diastolic contractions at 120 and 80 Hz, etc.). Therefore, a 10 Hz low pass filter was applied based on research of human movement and motion capture that demonstrates that normal physiologic movement happens at those lower frequencies [20,21].

### 3.3. Force Measurements

**Figure 7:**
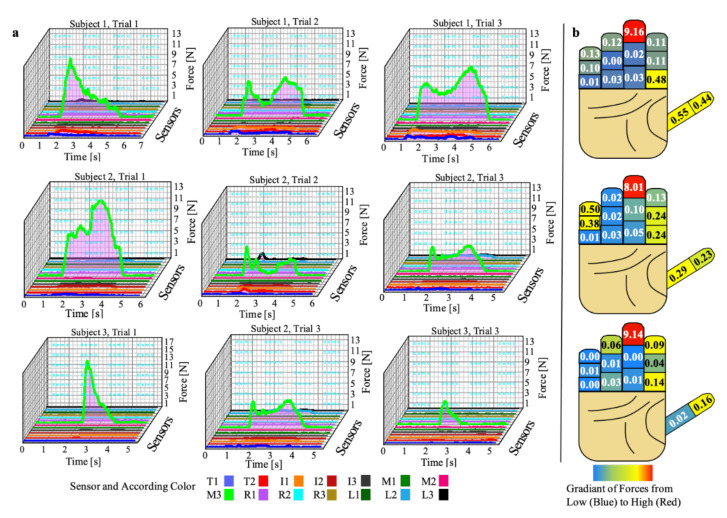
Can grasp results and analysis: (**a**) graphs of force during can grasp task with three subjects and three trials and (**b**) average maximum value during those three trials with a heat map of forces represented at each phalanx.

**Figure 8:**
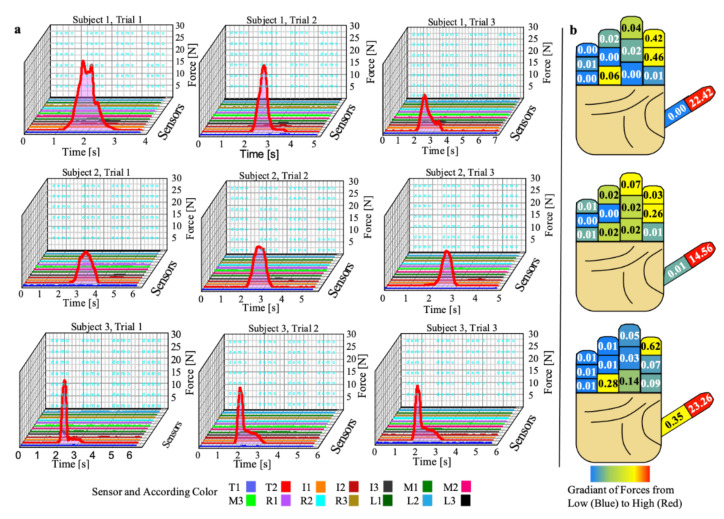
Key turn results and analysis: (**a**) graphs of force during key turn task with three subjects and three trials and (**b**) average maximum value during those three trials with a heat map of forces represented at each phalanx.

Figure 7 and Figure 8 illustrate the results of force measurements for each sensor in each panel corresponding to different participants and trials. Maximal forces were captured and recorded by choosing the frame with the largest force value during the performance of these tasks. Capturing data with the Codamotion system at 200 Hz allowed for precision when attempting to determine the greatest force. In the can grasp task, for each subject and trial the largest forces were observed at the middle distal phalanx between 8 and 9 N, on average. Meaningful but much smaller forces were noted at the thumb and index fingers. As can be seen in Participant 2 (second row), there were also small but meaningful forces noted at the little finger. A common occurrence was the lack of force noted at the little finger proximal phalanx, ring finger proximal, middle, and distal phalanges, and middle finger proximal and distal phalanges.

In the key turn tasks, the largest forces occurred at the distal phalanx of the thumb. The largest forces were a bit more variable than the can grasp task with a range between 14.56 and 23.26 N. Minimal forces occurred at the little, ring, and middle fingers. Each participant did demonstrate a meaningful but much smaller force value at the index finger. However, there was some variability in which phalange(s) produced that greater force. Comparing the participants, Participant 3 demonstrated greater force at the distal index phalanx, whereas Participant 2 demonstrated greater force at the index middle phalanx. In contrast, Participant 1 demonstrated greater forces at both the index middle and distal phalanges.

## 4. Discussion

The results reveal relatively similar maximum values for the can grasp task at the distal middle phalanx between participants. Those findings are somewhat expected since the middle finger is the longest and creates the greatest amount of torque in digits two to five. An expectation is that the midpoint of the can is in the middle of the hand. With this line of thinking, it makes sense that more control of the can is established by applying more force at the center of the can with the middle finger. However, there is an expectation for an equal and opposite reactionary force on the can from another spot on the hand. This force is necessary to create a sufficient frictional force that would prevent the can from slipping in the hand. In each of the participants, only minimal forces were recorded at other phalanges. Those summed forces of the other fingers did not equal the large force produced by the middle distal phalanx. This leads to two possible reasons for the lack of reaction force. First, the glove did not have enough sensors to accurately measure all the forces. There is an expectation that the hand is in a spherical grasp position when grasping the can. Therefore, the can would be in contact with the metacarpals/palm of the hand, as well as the phalanges. Since there were no sensors placed on the metacarpals, it needs to be considered that a portion of the reactional force may have occurred at the palm of the hand.

Another possible reason for the missing reaction forces is related to the specific coverage of the sensors on the phalanges, as the sensors have a diameter of 0.375 inches. The small size of the sensors did not completely cover the full palmar surface area on each of the phalanges. Due to the curvature and size of the phalanges, the radial and ulnar sides of the phalanges (portions of the fingers between the palmar and dorsal portions of the phalanges) are not covered. Reviewing the second participant’s results, compared to the first and third participants’, led to some insight regarding this problem. The second participant demonstrated small but meaningful forces at the little finger. However, meager forces were noted at the little finger during the first and third participants’ trials. It can be suspected that the forces produced by the first and third participants were not captured due to the placement of the can in the hand and lack of contact between the sensors and the can.

When exploring the key turn task results, a greater maximal force was observed in this experiment compared to the can task. This is somewhat expected due to the small size of the key. Because the key is so small, there is a smaller surface area to focus the force and provide the necessary friction that prevents the key from sliding in the hand. However, compared to the can task, there is a wide range of maximal forces between 14.56 N and 23.26 N. There may exist a few explanations why a wide range of forces existed during this experiment. First, the second participant may have utilized a different grasp type compared to the other two participants. It was hypothesized in this experiment that the participant would either use a lateral pinch or tip-to-tip pinch. Therefore, it was expected that the thumb and index would create equal and opposite reactional forces for the lateral pinch. As for tip-to-tip, the index and middle distal phalanges would create an equal and opposite reactional force to the thumb. The lack of forces recorded at those sensors infers that the current sensors did not properly capture the full force. If the participant utilized a lateral pinch, the radial side of the second finger would most likely register greater forces. If the tip-to-tip method was employed by the participant, the key was more than likely manipulated distally to the placement of the sensors in the glove.

Other limitations that possibly resulted in the omission of recorded forces are related to the fabric of the glove and the membrane of the sensor. Although the membrane of the sensor was relatively small (0.0625-inch thickness), the lack of sensory input between the fingers and the object may have altered the method of the participant manipulating the object. Similar to the membrane of the sensor, the fabric may have caused the participant to change their normal method of manipulating the object secondary to altered sensory feedback when grasping the object. The fabric also contained greater flexibility and a decreased frictional coefficient compared to human skin. Both of these properties could result in an altered grasp technique when wearing the glove compared to not wearing the glove and manipulating both objects. If more detailed force analysis is desired on a specific object, placing force sensors on both gloves and objects might provide a correlation of grasping forces from the hand as well as the object.

## 5. Conclusions

A force glove with FlexiForce sensors placed at each of the 14 phalanges was developed and tested to capture forces produced by the hand while performing a couple of ADLs. In this experiment, forces as large as 23.56 N were observed when grasping and turning a key. Smaller but still meaningful forces of approximately 9 N were observed when grasping a 12-ounce can. This information can assist in determining the minimal forces that an actuator will have to provide to a specific joint of the hand when modeling hand prosthetics and orthotics for ADLs. This information will also be useful when building algorithms to successfully perform an activity such as a can grasp. In spite of a limited participant sample size, this work still provides insight toward human biomimetics and additional studies on assessing hand forces during ADLs. The 9 N of force can provide a feedforward metric to the can grasp algorithm of an exoskeleton with actuators. Once the glove grasps the can, sensors on the glove may determine compressive or shear strain to determine if the user is deforming (crushing the can) or if the can is sliding through the hand. In this scenario, the actuator would either decrease the force (to prevent the user from crushing the can) or increase the force of the actuator to create more friction and prevent the can from sliding through the user’s hand.

As mentioned above, there were limitations in force capture due to sensor size, surface area coverage of the sensors, and fabric of the glove. Therefore, further work in this region would require the creation of a force sensor that covers a larger surface area of the fingers and palm of the hand. A sensor with grid patterns [22] would be a better method of capturing forces and would be more descriptive to the regions where the force is applied. The materials that would be used to create this grid pattern sensor should also more closely mimic the compression (Young’s Modulus) and shear properties of human skin. The sensory feedback to the user should also be considered when fabricating this device because that can alter the grip method.
